# Genetic polymorphisms of *IL17A* associated with Chagas disease: results from a meta-analysis in Latin American populations

**DOI:** 10.1038/s41598-020-61965-5

**Published:** 2020-03-19

**Authors:** Mariana Strauss, Miriam Palma-Vega, Desiré Casares-Marfil, Pau Bosch-Nicolau, María Silvina Lo Presti, Israel Molina, Clara Isabel González, Chagas Genetics CYTED Network, Patricia A. Paglini, Alejandro G. Schijman, Carlos Robello, Luis E. Echeverría, Gilberto Vargas-Alarcón, José E. Calzada, Mercedes Fernández-Mestre, Manuel Fresno, Maria Jesus Pinazo, Javier Martín, Marialbert Acosta-Herrera

**Affiliations:** 1 Centro de Estudios e Investigación de la Enfermedad de Chagas y Leishmaniasis, FCM, INICSA-CONICET-UNC, Córdoba, Argentina; 2 Instituto de Parasitología y Biomedicina López-Neyra, IPBLN-CSIC Granada, España; 3 Unidad de Medicina Tropical y Salud Internacional Hospital Universitari Vall d’Hebron, PROSICS, Barcelona, España; 4 GIEM, Universidad Industrial de Santander, Bucaramanga, Colombia; 5 Laboratorio de Biología Molecular de la Enfermedad de Chagas, INGEBI-CONICET, Buenos Aires, Argentina; 6 Laboratorio de Interacción Hospedero-Patógeno -UBM, Instituto Pasteur de Montevideo, Montevideo, Uruguay; 7 Clínica de insuficiencia cardíaca y trasplante de corazón, Fundación Cardiovascular de Colombia, Floridablanca, Colombia; 8 Departamento de Biología Molecular, Instituto Nacional de Cardiología Ignacio Chávez, México, México; 9 Instituto Conmemorativo Gorgas de Estudios de la Salud (ICGES), Panamá, Panamá; 10 Laboratorio de Fisiopatología, Centro de Medicina Experimental, Instituto Venezolano de Investigaciones Científicas, Caracas, 21827 Venezuela; 11 Departamento de Biología Molecular, Centro de Biología Molecular Severo Ochoa, Universidad Autónoma de Madrid, Madrid, España; 12 ISGlobal, Hospital Clínic, Universitat de Barcelona, Barcelona, España

**Keywords:** Genetic association study, Parasitic infection

## Abstract

Genetic factors and the immunologic response have been suggested to determine the susceptibility against the infection and the outcome of Chagas disease. In the present study, we analysed three *IL17A* genetic variants (rs4711998, rs8193036 and rs2275913) regarding the predisposition to *Trypanosoma cruzi* infection and the development of chronic Chagas cardiomyopathy (CCC) in different Latin American populations. A total of 2,967 individuals from Colombia, Argentina, Bolivia and Brazil, were included in this study. The individuals were classified as seronegative and seropositive for *T. cruzi* antigens, and this last group were divided into asymptomatic and CCC. For *T. cruzi* infection susceptibility, the *IL17A* rs2275913*A showed a significant association in a fixed-effect meta-analysis after a Bonferroni correction (P = 0.016, OR = 1.21, 95%CI = 1.06–1.41). No evidence of association was detected when comparing CCC *vs*. asymptomatic patients. However, when CCC were compared with seronegative individuals, it showed a nominal association in the meta-analysis (P = 0.040, OR = 1.20, 95%CI = 1.01–1.45). For the *IL17A* rs4711998 and rs8193036, no association was observed. In conclusion, our results suggest that *IL17A* rs2275913 plays an important role in the susceptibility to *T. cruzi* infection and could also be implicated in the development of chronic cardiomyopathy in the studied Latin American population.

## Introduction

Chagas disease caused by the protozoan *Trypanosoma cruzi* is a parasitic infection endemic in Latin American countries, which is nowadays increasingly becoming a global health problem, due to migration to non-endemic areas^1,2^. Around 6 to 7 million people are estimated to be infected worldwide, mostly in Latin America^1,3^. During the acute phase of the disease, the increase of parasitic load induces an inflammatory process for the control of the pathogen^4^. Ten to thirty years after infection, around 30 to 40% of chronically infected patients can develop cardiomyopathy or/and megaviscera. The symptoms include cardiac conduction abnormalities, myocardial contractile dysfunction, arrhythmias, dysphagia, regurgitation, and severe constipation, among others. The cardiac involvement is the most frequent manifestation of the disease^5,6^. The characteristics of this phase vary in different patients and in different regions of the endemic area^7^.

After the infection with *T. cruzi*, interleukin-17A (IL-17A) is produced by T helper 17 (Th17) cells, subset of CD4+ T cells, and innate lymphoid cells^8,9^. More recently, it has been described that B cells are also an important source of IL-17A and IL-17F, produced as well after *T. cruzi* infection^10,11^. In response to the infection, IL17-A, induces a rapid proinflammatory cascade of chemokines and cytokines that facilitates the recruitment and activation of neutrophils and monocytes required for the early control of the pathogen by the immune system^12,13^. In the chronic phase of the disease, several studies suggest that the clinical progression of Chagas cardiomyopathy involves the overexpression of IL-17 by Th17 cells and B cells^14,15^.

It’s well known that, genetic factors and immunologic response may determine the susceptibility against the infection and the outcome of Chagas disease^16–19^. Thus, polymorphisms in genes encoding cytokines may influence the level of cytokines production and, consequently, cause different immunological responses^20–22^. Specifically, *IL17A* polymorphisms, located in the promoter region of the gene, have been associated with plasma IL-17A levels in cell cultures^20^ and in healthy infants^23^. Several studies have found associations between the *IL17A* gene polymorphisms with infectious diseases, such as, cutaneous leishmaniasis^24^, brucellosis^25^, and tuberculosis^26^.

A previous study performed, by our group, in a Colombian cohort, observed nominal significant associations between variants of *IL17A* gene with the susceptibility to *T. cruzi* infection and with the development of chronic cardiomyopathy^27^. An additional study conducted in the South and Southeast regions of Brazil also found an association with *IL17A* and *IL17F* variants and the susceptibility to the development of chronic Chagas cardiomyopathy^28^.

Given the limited information about the role of *IL17A* gene variants in Chagas disease, this study aimed to analyse the association of three *IL17A* genetic variants with the predisposition to *T. cruzi* infection, the development of chronic cardiomyopathy and chronic Chagas cardiomyopathy, in different Latin American populations.

## Results

A total of 2,967 patients were included in the study. The demographic characteristics of the studied cohorts are reported in Table 1.

**Table 1:** Demographic characteristics of patients included in the present study classified by Chagas disease serology and symptoms.

	Seropositive		Seronegative	Total
	CCC	ASY		
Sex (% males)	40%*	31%*	36%**	
Median age, yr (P25–P75)	60 (51–68)*	48 (41–57)*	46 (31–62)**	
Colombian	576	361	640	1,577
Argentinian	182	90	78	350
Bolivian	100	530	—	630
Brazilian^28^	212	48	150	410
Total	1,070	1,029	868	2,967

CCC: chronic Chagas cardiomyopathy. ASY: asymptomatic.

*Data from the Colombian, Argentinian and Bolivian cohorts.

**Data from the Colombian and Argentinian cohorts.

The three single nucleotide polymorphisms (SNPs) of *IL17A* gene: rs4711998, rs8193036 and rs2275913, selected for this study, were in Hardy-Weinberg equilibrium in all the analysed cohorts (P > 0.01). The genotyping success rate was over 95% and the allele frequencies in all cases were similar to those described for the Americans sub-populations of the 1000 Genomes Project phase III (http://www.1000genomes.org)^29^ (Table S1).

### *T. cruzi* infection susceptibility

The allelic and genotypic frequencies of seronegative and seropositive individuals from the Colombian cohort were compared in Tables S3–1 The minor allele frequency (MAF), in rs8193036*C was higher in seronegative than in seropositive individuals, being nominally significant after the adjustment by sex and age [P = 0.043, odds ratio (OR) = 0.83, 95% confidence interval (CI) = 0.70–0.99]. No associations between the three *IL17A* genetic variants and susceptibility to *T. cruzi* infection were found after performing logistic regression adjusted by sex and age in the Argentinian cohort (Tables S3–2**)**. In contrast, interestingly, the SNP *IL17A* rs2275913*A, that was studied in a Brazilian cohort, was found statistically significant for the risk to *T. cruzi* infection^28^. Furthermore, a meta-analysis combining each individual cohort (Colombian, Argentinian and Brazilian) was performed (Table 2). The *IL17A* rs2275913*A allele effect was consistent in the three cohorts and the association improved after the meta-analysis, showing statistically significant results (P = 0.016, OR = 1.21, 95% CI = 1.06–1.41, under a fixed-effects meta-analysis) after Bonferroni correction. No association was observed for the *IL17A* rs4711998 and rs8193036.

**Table 2:** Meta-analysis of *IL17A* variants, Latin American cohorts for *T. cruzi* infection susceptibility (seropositive *vs*. seronegative individuals).

	Colombian cohort		Argentinian cohort		Brazilian cohort		Meta-analysis	
SNP	OR (L95-U95)	P	OR (L95-U95)	P	OR (L95-U95)	P	OR (L95-U95)	P
rs4711998*A	0.94 (0.78–1.14)	0.528	1.38 (0.90–2.12)	0.143	—	—	0.99 (0.84–1.17)	0.946
rs8193036*C	0.83 (0.70–0.99)	0.043	1.34 (0.89–20.2)	0.164	—	—	0.89 (0.76–1.05)	0.169
rs2275913*A	1.16 (0.95–1.4)	0.136	1.07 (0.67–1.69)	0.793	1.46 (1.05–2.05)	0.032	1.21 (1.06-1.41)	**0.016**

Total number of individuals: rs4711998 and rs8193036: seropositive, n = 1209 and seronegative, n = 718; rs2275913: seropositive, n = 1469 and seronegative, n = 868. OR: odds ratios, L95-U95: confidence intervals of 95% L: lower limit; U: upper limit. Significant P value is shown in bold. Significant association based on the Bonferroni correction P < 0.017.

### Chronic chagas cardiomyopathy susceptibility

To understand the genetic basis of chronic Chagas cardiomyopathy, we compared CCC and asymptomatic patients. The allelic frequencies of *IL17A* variants in patients from the Colombian, Argentinian and Bolivian cohorts were not statistically significant after the logistic regression adjusted by sex and age (Tables S4–1–3**)**, consistent with previous findings in the Brazilian cohort^28^. Moreover, no significant associations were detected for the available SNPs when the meta-analysis was performed combining these cohorts (Table 3).

**Table 3:** Meta-analysis of *IL17A* variants, Latin American cohorts for chronic Chagas cardiomyopathy susceptibility (CCC *vs*. asymptomatic patients).

	Colombian cohort		Argentinian cohort		Bolivian cohort		Brazilian cohort		Meta-analysis	
SNP	OR (L95-U95)	P	OR (L95-U95)	P	OR (L95-U95)	P	OR (L95-U95)	P	OR (L95-U95)	P
rs4711998*A	0.86 (0.67–1.11)	0.259	1.08 (0.69–1.68)	0.751	0.96 (0.65–1.41)	0.831	—	—	0.92 (0.76–1.11)	0.396
rs8193036*C	0.92 (0.72–1.18)	0.526	0.74 (0.49–1.29)	0.164	1.18 (0.85–1.62)	0.319	—	—	0.95 (0.80–1.14)	0.616
rs2275913*A	0.8 (0.62–1.02)	0.081	0.72 (0.43–1.21)	0.217	1.14 (0.75–1.71)	0.543	1.21 (0.74–1.99)	0.463	0.89 (0.74–1.07)	0.232

Total number of individuals: rs4711998 and rs8193036: CCC, n = 858 and asymptomatic, n = 981; rs2275913: CCC, n = 1070 and asymptomatic, n = 1029. OR: odds ratios, L95-U95: confidence intervals of 95% L: lower limit; U: upper limit.

Finally, in order to evaluate the possible association between *IL17A* genetic variants and chronic cardiomyopathy we compared CCC patients and seronegative individuals, as previously performed^27,28^. In the Colombian and Argentinian cohort (Tables S5–1 and 2**)** and no associations were found after applying logistic regression adjusted by sex and age. In contrast, a previous study, in the Brazilian cohort the *IL17A* rs2275913*A allele was nominally significant^28^. As can be observed in Table 4 the *IL17A* rs2275913 allele effect was consistent in the Colombian, Argentinian and Brazilian cohorts, and the association with chronic cardiomyopathy susceptibility improved after the meta-analysis showing nominally statistical differences (P = 0.040, OR = 1.20, 95% CI = 1.01–1.45, under a fixed-effects meta-analysis). These results suggest that rs2275913*A allele was associated with the risk of cardiomyopathy in the analysed population.

**Table 4:** Meta-analysis of *IL17A* variants, Latin American cohorts for chronic cardiomyopathy susceptibility (CCC *vs*. seronegative individuals).

	Colombian cohort		Argentinian cohort		Brazilian cohort		Meta-analysis	
SNP	OR (L95-U95)	P	OR (L95-U95)	P	OR (L95-U95)	P	OR (L95-U95)	P
rs4711998*A	0.93 (0.75–1.16)	0.541	1.47 (0.92–2.37)	0.109	—	—	1.02 (0.82–1.23)	0.927
rs8193036*C	0.84 (0.67–1.05)	0.133	1.21 (0.78–1.88)	0.389	—	—	0.91 (0.74–1.10)	0.323
rs2275913*A	1.14 (0.90–1.44)	0.298	0.99 (0.60–1.61)	0.955	1.52 (1.08–2.15)	0.021	1.20 (1.01–1.45)	0.040

Total number of individuals: rs4711998 and rs8193036: CCC, n = 758 and seronegative, n = 718; rs2275913: CCC, n = 970 and seronegative, n = 868. OR: odds ratios, L95-U95: confidence intervals of 95% L: lower limit; U: upper limit.

### *In silico* functional characterization of *IL17A* gene variants

Since the *IL17A* rs2275913 showed a statistical association with the risk to *T. cruzi* infection and the development of chronic cardiomyopathy, we perform an *in silico* functional analysis of this SNP and the ones in high LD (R^2^ ≥ 0.8) on peripheral mononuclear blood in American population from the 1000 Genomes Project (Table 5**)**. The annotation indicates that these SNPs map in enhancer regions and marks of histone modifications (H3K4me1, H3K4me3, H3K27ac and chromatin marks), potentially modulating gene expression.

**Table 5:** Functional annotation.

Position^a^	SNPs	R^2^	Functionality	MAF (AMR)	eQTL	Chromatin states^b^	Chromatin states^c^	H3K4me1	H3K4me3	H3K27ac
chr6:52051033	rs2275913	1	Intergenic variant	25%	—	Flank	Promoter	Enhancer	Promoter	Enhancer
chr6:52087034	rs11966760	0.82	Intergenic variant	24%	PAQR8	Enhancer	Promoter	Enhancer	Promoter	Enhancer
chr6:52056386	rs16882180	0.8	Intergenic variant	25%	PAQR8	—	—	Enhancer	Promoter	—

Regulatory chromatin states and histone modifications for *IL17A* rs2275913 and SNPs in high LD (R^2^  ≥  0.8).

Functional annotation from mononuclear peripheral blood specifically primary T helper 17 cells.

^a^According to National Center for Biotechnology Genome Reference Consortium NCBI build GRCh37.

^b^Core 15-state model.

^c^Chromatin states: 25-state model using 12 imputed marks.

H3K4me1: Histone H3 lysine 4 mono-methylation, H3K4me3: Histone H3 lysine 4 tri-methylation, H3K27ac: Histone H3 lysine 27 acetylation.

MAF: Minor Allele Frequency. AMR: American.

## Discussion

Association studies offer a potentially powerful approach to identify genetic variations that are involved in the immunopathogenesis of Chagas disease^16–19^. However, individual genetic association studies frequently have limitations and the results may be specific to the population of the study. The meta-analysis approach has been proposed to resolve these limitations, to increase the power of statistical analyses^30,31^ and to reach to more conclusive results in order to improve our understanding of the genetic basis underlying Chagas disease. In this study, three *IL17A* genetic variants were analysed in four Latin American populations, and our results evidenced the implication of rs2275913 associated with the risk to *T. cruzi* infection and the development of chronic cardiomyopathy, in Colombian, Argentinian and Brazilian population.

In the early stages of the infection, the IL-17A is a crucial cytokine secreted by a wide range cell types such as Th17, B cells, innate lymphoid cells, CD4+, CD8+, gamma-delta T and invariant NKT^10-13,32^. The rs2275913, which was associated with the risk to *T. cruzi* infection in the Latin American population studied, is a functional polymorphism that modifies the binding of the transcriptional nuclear factor of activated T cells (NFAT) in the IL-17A promoter. Moreover, as observed in the *in silico* analysis, the associated variant is located in promoter histone marks, potentially modulating gene expression. In addition, it has been demonstrated that the substitution of the G by an A allele in the IL17A rs2275913 gene promoter was significantly associated with autoimmune diseases and cancer^33–36^. However, controversial results have been reported regarding the levels of IL-17A in serum, where the A allele was associated with a higher^20,36,37^, lower^38,39^ or no significant^40^ levels of transcription and synthesis of the protein. In this work, we could hypothesized that the individuals who carry the A allele in the *IL17A* rs2275913 would be more susceptibility to the *T. cruzi* infection, probably due to a variation in the gene expression and therefore lower IL-17A production, which would impede a rapid proinflammatory activation of chemokines and cytokines for the resolution of *T. cruzi* infection^12,13^. However, further studies are required to understand the complexity of *IL17A* rs2275913 polymorphism functional effect.

Several studies showed that IL-17A has an important immunomodulatory role in the chronic phase of the disease^8,14,15,41^. IL‐17 expression by Th17 cells and B cells were found in patients with cardiac involvement more frequently, compared to asymptomatic patients, correlating with worse cardiac function^14^; IL-17 exacerbated produce a proinflammatory environment in Chagas severe heart disease^42,43^. However controversially, others groups suggested a protective role of IL‐17 and Th17 in the chronic cardiac form^44,45^. Regarding *IL17A* genetic variants, in our study, no significant association was detected when CCC and asymptomatic patients from Colombia, Argentina, Bolivia and Brazil were compared. This lack of association could be a consequence of an insufficient statistical power (Table S1) or genetic heterogeneity among the studied cohorts. The lack of replication may occur if the studied polymorphism is not the causal variant but is rather in LD with it. LD patterns depend on the genetic background of the founder population and population history, always challenging in Latin American population due to their genetic diversity and recent admixture^46–48^.

Interestingly, at the present time IL-17A have become a relevant drug target in various forms of autoimmune and inflammatory diseases, mainly as negative modulators of the secreted protein^13,49^. Two antibodies are currently in Phase IV of drug development for the treatment of immune system diseases, namely, Secukinumab and Ixekizumab (Anatomical Therapeutic Chemical [ATC] code L04AC10 and L04AC13, respectively). Given the role of IL-17A as a key cytokine in the pathogenesis of Chagas disease, the opportunities for drug repurposing becomes very important for this neglected disease, as there are only two treatments available: Benznidazole and Nifurtimox, with high rates of adverse effects and treatment withdrawal^50,51^.

In conclusion, in this work, we found an association of *IL17A* rs2275913 with Chagas disease in a large cohort composed of different Latin American countries, validating previous findings^27,28^. Finally, further studies on this gene accounting for functional analyses and heterogeneity among populations, would be necessary for the complete understanding of *IL17A* polymorphisms in Chagas disease.

## Materials and Methods

### Study design and patient population

A candidate-gene case-control study was performed in Colombian, Argentinian and Bolivian cohorts in order to replicate previous findings^27,28^. In addition, to improve the statistical power of the study a meta-analysis was performed combining the results from all the available cohorts.

A total of 2,967 individuals from Latin American countries (Argentina, Bolivia Colombia and Brazil) were studied. In all cohorts, patients were classified as seropositive for *T. cruzi* antigens (n = 2,099) and seronegative (n = 868) on the basis of results of at least 2 of 3 independent tests. Among seropositive individuals, based on electrocardiographic, echocardiographic, chest X-ray and clinical findings, patients were classified as chronic Chagas cardiomyopathy (CCC, n = 1,070) and asymptomatic (ASY, n = 1,029).

#### Colombian cohort

A total of 406 Colombian individuals from the same population as the study by Leon Rodriguez *et al*.^27^ were recruited by the health care team from the Industrial University of Santander and Cardiovascular Foundation from Colombia. In order to increase the sample size, these individuals were included with the previously published Colombian cohort, making a total of 1,577 individuals. From this, 937 were classified as seropositive for *T. cruzi* antigens and 640 were classified as seronegative (according to the serological tests: recombinant antigen ELISA and commercial indirect hemagglutination test). Subsequently, and after a clinical evaluation, an electrocardiogram (ECG) and an echocardiogram (Echo) were recorded to detect any conduction and/or structural alteration in seropositive patients. Based on complementary tests and clinical findings, seropositive patients were classified as CCC = 576 and ASY = 361. The mean age of participants was 45.55 ± 17.19 years for seronegative individuals, CCC = 61.44 ± 12.82 and ASY = 51.90 ± 14.18. The sex distribution was 58% female and 42% male^52^.

#### Argentinian cohort

A total of 350 Argentinian individuals from an endemic region for Chagas disease (Cordoba province) were included in this study. The study subjects were recruited from the National Hospital of Clinics and Sucre Clinic, Cordoba city. The population in this region of Argentina is a homogeneous mixture, with no specific concentration of any ethnicity. All participants underwent a serological diagnosis for *T. cruzi* infection by means of the enzyme-linked immunosorbent assay (ELISA) that uses recombinant antigen and a commercial indirect hemagglutination test. According to the results of these tests, 272 individuals were classified as seropositive for *T. cruzi* antigens and 78 were classified as seronegative. Based on the results of clinical findings, ECG and Echo, seropositive patients were classified as CCC, n = 182 and ASY, n = 90. The mean age of participants was 53.82 ± 16.53 years for seronegative individuals, 49.30 ± 13.65 for asymptomatic individuals and 60.14 ± 10.16 for chronic Chagas cardiomyopathy patients. The sex distribution was 71% female and 29% male^52^.

#### Bolivian cohort

A total of 630 Bolivian individuals residents in Barcelona, Spain were recruited from the Infectious Diseases Department of the Vall d’Hebron University Hospital. In this cohort only seropositive patients were classified as CCC, n = 100 and ASY, n = 530 based on the results of clinical findings, ECG and Echo. The sex distribution was 69% female and 31% male. The mean age of the participants was ASY: 46.93 ± 9.49 CCC: 50.71 ± 9.41^52^.

#### Brazilian cohort

Data from 410 Brazilian individuals drawn from Reis *et al*. 2017, originally from South and Southeast regions of Brazil were included in the meta-analysis^28^. From this, 260 were classified as seropositive for *T. cruzi* antigens and 150 were classified as seronegative. Based on complementary tests and clinical findings, seropositive patients were classified as CCC = 212 and ASY = 48.

### Ethics statement

The study was accepted by the Ethics Committees from the Industrial University of Santander and Cardiovascular Foundation, Colombia [Act No. 15/2005]; the Vall d’Hebron University Hospital, Barcelona, Spain and the National Hospital of Clinics [PR (AMI) 297/2016], National University of Cordoba, Argentina [CIEIS HNC 118/2012 and 2/16/2017]. Written informed consent was obtained from all subjects prior to participation. The research protocols followed the principles of the Declaration of Helsinki and informed consent was obtained from all individual participants included in the study.

### Selected polymorphisms and genotyping

Three SNPs of *IL17A* gene (rs4711998, rs8193036 and rs2275913) were selected for this study. These SNPs were previously assessed in Chagas disease in a Colombian cohort and in a Brazilian cohort^28^. These SNPs are independent intergenic variants mapping to the promoter region of *IL17A*^53^ (linkage disequilibrium [LD], r^2^ < 0.2 estimated using LDlink website tool [https://ldlink.nci.nih.gov/?tab=ldmatrix]).

Genomic DNA from blood samples was isolated following standard procedures and the genotyping was performed using TaqMan assays (Applied Biosystems, Foster City, California, USA) on a real-time PCR system (7900HT Fast Real-Time PCR System), SNPs were determined by TaqMan 5´ allelic discrimination assay method performed by Applied Biosystems^52^.

### Statistical analysis

For the candidate gene study, the statistical analyses were performed with the software Plink V1.9 (https://www.cog-genomics.org/plink2)^54^. Deviance from Hardy-Weinberg equilibrium was determined at the 1% significance level in all groups of individuals. Individuals that did not achieved an SNP completion rate of 95% were filtered out. To test for possible allelic association, logistic regression model and Fisher’s exact test were assessed in seronegative *vs*. seropositive individuals and asymptomatic *vs*. chronic Chagas cardiomyopathy individuals, using age and sex as covariates; and odds ratios (OR) and 95% confidence intervals (CI) were calculated. P-values lower than 0.05 were considered as statistically significant.

To assess the consistency of effects across the cohorts, a meta-analysis was performed with METASOFT (http://genetics.cs.ucla.edu/meta/) based on inverse-variance-weighted effect size. Heterogeneity across studies was assessed using the Cochran’s Q statistic (Q test P < 0.05) and *I*^2^ heterogeneity index^55^. A fixed-effects model was applied for those SNPs without evidence of heterogeneity (Cochran’s Q test P  >  0.05), and a random-effects model was applied for SNPs displaying heterogeneity of effects between studies (Cochran’s Q test P  ≤  0.05). The significance threshold for the meta-analyses was estimated based on the Bonferroni correction (0.05/3 = 0.017)^56^.

The statistical power of the studies was estimated with the Power Calculator for Genetic Studies 2006 (CaTS) software (Tables S1 and S2) (http://www.sph.umich.edu/csg/abecasis/CaTS/)^57^.

### *In silico* functional characterization of *IL17A* gene variants

Evaluation of functionality of the statistically significant associated SNP with Chagas disease, was performed with the online software HaploReg v4.1^58^. (https://pubs.broadinstitute.org/mammals/haploreg/ haploreg.php) based on empirical data from the ENCODE project (http://www.genome.gov/encode/). Specifically, we focused our attention on experiments performed on blood in the American population. Moreover, updated information related to expression Quantitative Trait Loci (eQTL) were inspected for *IL17A* rs2275913 and for SNPs in high LD (R^2^  ≥  0.8) (Table 5).

## Supplementary information

Supplementary information.

Supplementary information 2.

Supplementary information 3.

Supplementary information 4.

Supplementary information 5.

## Data Availability

All relevant data are within the paper and its Supporting Information files.
