# Supplementary material for: Genetic polymorphisms of IL17A associated with Chagas disease: results from a meta-analysis in Latin American populations
Source: Sci Rep. 2020 Mar 19;10:5015. doi: 10.1038/s41598-020-61965-5 (PMC7081280; doi:10.1038/s41598-020-61965-5)
Supplement: Supplementary file 1 — Supplementary information. [file 41598_2020_61965_MOESM1_ESM.docx]

*Genetic polymorphisms of IL17A associated with Chagas disease: results from a meta-analysis in Latin American populations*

Mariana Strauss, Miriam Palma-Vega, Desiré Casares-Marfil, Pau Bosch-Nicolau, María Silvina Lo Presti, Israel Molina, Clara Isabel González, Chagas Genetics CYTED Network, Javier Martín, Marialbert Acosta-Herrera

**Table S1. Statistical power calculation considering different effect sizes: Candidate gene approach**

Table S1-1

|  |  |  | *Colombia* | | |
| --- | --- | --- | --- | --- | --- |
| SNP | MAF | OR | *T. cruzi* infection (937/640)* | Chronic Chagas cardiomyopathy. CCC *vs.* asymptomatic (576/361) | Chronic Chagas cardiomyopathy. CCC *vs.* seronegative (576/640) |
| rs4711998 | 25% | OR=1.30 | 91% | 71% | 83% |
|  |  | OR=1.20 | 62% | 41% | 52% |
|  |  | OR=1.10 | 22% | 17% | 18% |
| rs8193036 | 24% | OR=1.30 | 90% | 70% | 82% |
|  |  | OR=1.20 | 61% | 40% | 51% |
|  |  | OR=1.10 | 21% | 16% | 17% |
| rs2275913 | 22% | OR=1.30 | 88% | 68% | 80% |
|  |  | OR=1.20 | 79% | 38% | 49% |
|  |  | OR=1.10 | 19% | 14% | 15% |

The estimation was performed considering a Chagas disease prevalence of 1.44% in Colombia [1]. MAF: minor allele frequency.

The allele frequencies used were those described for the Americans sub-populations of the 1000 genomes phase III project (<http://www.1000genomes.org>).

Table S1-2

|  |  |  | *Argentina* | | |
| --- | --- | --- | --- | --- | --- |
| SNP | MAF | OR | *T. cruzi* infection (272/78)* | Chronic Chagas cardiomyopathy. CCC vs. asymptomatic (182/90) | Chronic Chagas cardiomyopathy. CCC *vs.* seronegative (182/78) |
| rs4711998 | 25% | OR=1.70 | 88% | 83% | 83% |
|  |  | OR=1.60 | 79% | 73% | 73% |
|  |  | OR=1.50 | 66% | 59% | 59% |
| rs8193036 | 24% | OR=1.70 | 87% | 82% | 82% |
|  |  | OR=1.60 | 78% | 72% | 72% |
|  |  | OR=1.50 | 65% | 59% | 59% |
| rs2275913 | 22% | OR=1.70 | 86% | 81% | 81% |
|  |  | OR=1.60 | 76% | 70% | 70% |
|  |  | OR=1.50 | 63% | 56% | 56% |

The estimation was performed considering a Chagas disease prevalence of 3.6% in Argentina [1]. MAF: minor allele frequency.

The allele frequencies used were those described for the Americans sub-populations of the 1000 genomes phase III project (<http://www.1000genomes.org>).

Table S1-3

|  |  |  | *Bolivia* |
| --- | --- | --- | --- |
| SNP | MAF | OR | Chronic Chagas cardiomyopathy (100/530)* |
| rs4711998 | 25% | OR=1.60 | 82% |
|  |  | OR=1.50 | 70% |
|  |  | OR=1.40 | 53% |
| rs8193036 | 24% | OR=1.60 | 81% |
|  |  | OR=1.50 | 68% |
|  |  | OR=1.40 | 52% |
| rs2275913 | 22% | OR=1.60 | 79% |
|  |  | OR=1.50 | 66% |
|  |  | OR=1.40 | 50% |

The estimation was performed considering a Chagas disease prevalence of 6.1% in Bolivia [1].

MAF: minor allele frequency.

The allele frequencies used were those described for the Americans sub-populations of the 1000 genomes phase III project (<http://www.1000genomes.org>).

*Analysis performed by using cases *vs.* controls individuals.

**Bibliography**

[1]. Word Health Organization/Department of control of neglected tropical diseases (2017) Integrating neglected tropical diseases in global health and development. 4th WHO report on neglect trop diseases. Geneva: World Health Organization.

[2]. Dias JC, Ramos AN Jr, Gontijo ED, Luquetti A, Shikanai-Yasuda MA, et al. (2016) Brazilian Consensus on Chagas Disease, 2015. Epidemiol Serv Saude 25: 7-86. doi: 10.5123/S1679-49742016000500002. PMID: 27869914
