# Supplementary material for: Genetic polymorphisms of IL17A associated with Chagas disease: results from a meta-analysis in Latin American populations
Source: Sci Rep. 2020 Mar 19;10:5015. doi: 10.1038/s41598-020-61965-5 (PMC7081280; doi:10.1038/s41598-020-61965-5)
Supplement: Supplementary file 2 — Supplementary information 2. [file 41598_2020_61965_MOESM2_ESM.docx]

*Genetic polymorphisms of IL17A associated with Chagas disease: results from a meta-analysis in Latin American populations*

Mariana Strauss, Miriam Palma-Vega, Desiré Casares-Marfil, Pau Bosch-Nicolau, María Silvina Lo Presti, Israel Molina, Clara Isabel González, Chagas Genetics CYTED Network, Javier Martín, Marialbert Acosta-Herrera

**Table S2. Statistical power calculation considering different effect sizes: Meta-analysis**

Table S2-1

|  |  |  | *Argentina, Colombia* | |
| --- | --- | --- | --- | --- |
| SNP | MAF | OR | *T. cruzi* infection (1209/718)* | Chronic Chagas cardiomyopathy. CCC *vs.* seronegative (758/718)* |
| rs4711998 | 25% | OR=1.30 | 95% | 90% |
|  |  | OR=1.20 | 71% | 62% |
|  |  | OR=1.10 | 25% | 21% |
| rs8193036 | 24% | OR=1.30 | 94% | 89% |
|  |  | OR=1.20 | 70% | 61% |
|  |  | OR=1.10 | 24% | 20% |

The estimation was performed considering an average of Chagas disease prevalence in Argentina (3.6%) and Colombia (1.44%): 2.52%. MAF: minor allele frequency.

The allele frequencies used were those described for the Americans sub-populations of the 1000 genomes phase III project (<http://www.1000genomes.org>).

Table S2-2

|  |  |  | *Argentina, Colombia, Brazil* | |
| --- | --- | --- | --- | --- |
| SNP | MAF | OR | *T. cruzi* infection (1469/868)* | Chronic Chagas cardiomyopathy. CCC *vs.* seronegative (970/868) |
| rs2275913 | 22% | OR=1.40 | 99% | 99% |
|  |  | OR=1.30 | 95% | 92% |
|  |  | OR=1.20 | 76% | 65% |
|  |  | OR=1.10 | 27% | 23% |

The estimation was performed considering an average of Chagas disease prevalence in Argentina (3.6%), Colombia (1.44%), and Brazil (2.4%) [2]: 2.48%. MAF: minor allele frequency. The allele frequency used were those described for the Americans sub-populations of the 1000 genomes phase III project (<http://www.1000genomes.org>).

Table S2-3

|  |  |  | *Argentina, Bolivia, Colombia* |
| --- | --- | --- | --- |
| SNP | MAF | OR | Chronic Chagas cardiomyopathy (858/981)* |
| rs4711998 | 25% | OR=1.30 | 96% |
|  |  | OR=1.20 | 72% |
|  |  | OR=1.10 | 26% |
| rs8193036 | 24% | OR=1.30 | 95% |
|  |  | OR=1.20 | 71% |
|  |  | OR=1.10 | 25% |

The estimation was performed considering an average of Chagas disease prevalence in Argentina, Colombia and Bolivia: 3.713%. MAF: minor allele frequency. The allele frequencies used were those described for the Americans sub-populations of the 1000 genomes phase III project (<http://www.1000genomes.org>).

Table S2-4

|  |  |  | *Argentina, Bolivia, Colombia, Brazil* |
| --- | --- | --- | --- |
| SNP | MAF | OR | Chronic Chagasic cardiomyopathy (1070/1029)* |
| rs2275913 | 22% | OR=1.30 | 97% |
|  |  | OR=1.20 | 74% |
|  |  | OR=1.10 | 27% |

The estimation was performed considering an average of Chagas disease prevalence in Argentina, Colombia, Bolivia and Brazil: 3.385%. MAF: minor allele frequency. The allele frequency used were those described for the Americans sub-populations of the 1000 genomes phase III project (<http://www.1000genomes.org>).

--

*Analysis performed by using cases *vs.* controls individuals.

**Bibliography**

[1]. Word Health Organization/Department of control of neglected tropical diseases (2017) Integrating neglected tropical diseases in global health and development. 4th WHO report on neglect trop diseases. Geneva: World Health Organization.

[2]. Dias JC, Ramos AN Jr, Gontijo ED, Luquetti A, Shikanai-Yasuda MA, et al. (2016) Brazilian Consensus on Chagas Disease, 2015. Epidemiol Serv Saude 25: 7-86. doi: 10.5123/S1679-49742016000500002. PMID: 27869914
