# Supplementary material for: Genetic polymorphisms of IL17A associated with Chagas disease: results from a meta-analysis in Latin American populations
Source: Sci Rep. 2020 Mar 19;10:5015. doi: 10.1038/s41598-020-61965-5 (PMC7081280; doi:10.1038/s41598-020-61965-5)
Supplement: Supplementary file 3 — Supplementary information 3. [file 41598_2020_61965_MOESM3_ESM.docx]

*Genetic polymorphisms of IL17A associated with Chagas disease: results from a meta-analysis in Latin American populations*

Mariana Strauss, Miriam Palma-Vega, Desiré Casares-Marfil, Pau Bosch-Nicolau, María Silvina Lo Presti, Israel Molina, Clara Isabel González, Chagas Genetics CYTED Network, Javier Martín, Marialbert Acosta-Herrera

**Table S3.** Genotype and allele distribution for *IL17A* variants in seronegative and seropositive individuals

**Table S3-1.** Colombian cohort

| SNP |  | A1\| A2 | Genotype N (%) | | | MAF | Allele test | | |
| --- | --- | --- | --- | --- | --- | --- | --- | --- | --- |
|  |  |  | 1\|1 | 1\|2 | 2\|2 |  | OR | (L95-U95) | P LogstReg |
| rs4711998 | Seronegative (621) | A\|G | 49 (7.89) | 217 (34.94) | 355 (57.16) | 25.36% | 0.94 | (0.78-1.14) | 0.528 |
|  | Seropositive (920) |  | 52 (5.65) | 332 (36.08) | 536 (58.26) | 23.70% |  |  |  |
| rs8193036 | Seronegative (621) | C\|T | 54 (8.69) | 229 (36.87) | 338 (54.42) | 27.13% | 0.83 | (0.70-0.99) | **0.043** |
|  | Seropositive (920) |  | 54 (5.87) | 317 (34.46) | 549 (59.67) | 23.10% |  |  |  |
| rs2275913 | Seronegative (621) | A\|G | 48 (5.22) | 294 (31.96) | 578 (62.83) | 19.00% | 1.16 | (0.95-1.4) | 0.136 |
|  | Seropositive (920) |  | 25 (4.03) | 186 (29.95) | 410 (66.02) | 21.20% |  |  |  |

1: minor allele | 2: major allele; alleles are showed in forward strand. MAF: minor allele frequency. OR: odds ratios, L95-U95: confidence intervals of 95% L: lower limit; U: upper limit. Values adjusted by sex and age.

**Table S3-2.** Argentinian cohort.

| SNP |  | A1\| A2 | Genotype. N (%) | | | MAF | Allele test | | |
| --- | --- | --- | --- | --- | --- | --- | --- | --- | --- |
|  |  |  | 1\|1 | 1\|2 | 2\|2 |  | OR | (L95-U95) | P LogstReg |
| rs4711998 | Seronegative (76) | A\|G | 4 (5.26) | 22 (28.95) | 50 (65.79) | 19.74 | 1.38 | (0.90-2.12) | 0.143 |
|  | Seropositive (272) |  | 21 (7.72) | 97 (35.66) | 154 (56.62) | 25.55 |  |  |  |
| rs8193036 | Seronegative (76) | C\|T | 5 (6.58) | 21 (27.63) | 50 (65.79) | 20.39 | 1.34 | (0.89-20.2) | 0.164 |
|  | Seropositive (272) |  | 29 (10.66) | 86 (31.62) | 157 (57.72) | 26.47 |  |  |  |
| rs2275913 | Seronegative (76) | A\|G | 4 (5.26) | 24 (31.58) | 48 (63.16) | 21.05 | 1.07 | (0.67-1.69) | 0.793 |
|  | Seropositive (272) |  | 7 (2.57) | 103 (37.87) | 162 (59.56) | 21.51 |  |  |  |

1: minor allele | 2: major allele; alleles are showed in forward strand. MAF: minor allele frequency. OR: odds ratios, L95-U95: confidence intervals of 95% L: lower limit; U: upper limit. Values adjusted by sex and age.
