# Supplementary material for: Genetic polymorphisms of IL17A associated with Chagas disease: results from a meta-analysis in Latin American populations
Source: Sci Rep. 2020 Mar 19;10:5015. doi: 10.1038/s41598-020-61965-5 (PMC7081280; doi:10.1038/s41598-020-61965-5)
Supplement: Supplementary file 4 — Supplementary information 4. [file 41598_2020_61965_MOESM4_ESM.docx]

*Genetic polymorphisms of IL17A associated with Chagas disease: results from a meta-analysis in Latin American populations*

Mariana Strauss, Miriam Palma-Vega, Desiré Casares-Marfil, Pau Bosch-Nicolau, María Silvina Lo Presti, Israel Molina, Clara Isabel González, Chagas Genetics CYTED Network, Javier Martín, Marialbert Acosta-Herrera

**Table S4.** Genotype and allele distribution for *IL17A* variants in seropositive (Asymptomatic) and Chronic Chagas cardiomyopathy (CCC) individuals

**Table S4-1.** Colombian cohort

| SNP |  | A1\| A2 | Genotype N (%) | | | MAF | Allele test | | |
| --- | --- | --- | --- | --- | --- | --- | --- | --- | --- |
|  |  |  | 1\|1 | 1\|2 | 2\|2 |  | OR | (L95-U95) | P LogstReg |
| rs4711998 | Asymptomatic (355) | A\|G | 21 (5.92) | 132 (37.18) | 202 (56.90) | 24.51% | 0.86 | (0.67-1.11) | 0.259 |
|  | CCC (565) |  | 31 (5.49) | 200 (35.40) | 334 (59.12) | 23.19% |  |  |  |
| rs8193036 | Asymptomatic (355) | C\|T | 26 (7.32) | 113 (31.83) | 216 (60.85) | 23.24% | 0.92 | (0.72-1.18) | 0.526 |
|  | CCC (565) |  | 28 (4.96) | 204 (36.11) | 333 (58.94) | 23.01% |  |  |  |
| rs2275913 | Asymptomatic (355) | A\|G | 21 (5.92) | 119 (33.52) | 215 (60.56) | 22.68% | 0.80 | (0.62-1.02) | 0.081 |
|  | CCC (565) |  | 27 (4.78) | 175 (30.97) | 363 (64.25) | 20.27% |  |  |  |

1: minor allele | 2: major allele; alleles are showed in forward strand. MAF: minor allele frequency. OR: odds ratios, L95-U95: confidence intervals of 95% L: lower limit; U: upper limit. Values adjusted by sex and age.

**Table S4-2.** Argentinian cohort

| SNP |  | A1\| A2 | Genotype N (%) | | | MAF | Allele test | | |
| --- | --- | --- | --- | --- | --- | --- | --- | --- | --- |
|  |  |  | 1\|1 | 1\|2 | 2\|2 |  | OR | (L95-U95) | P LogstReg |
| rs4711998 | Asymptomatic (90) | A\|G | 11 (12.22) | 24 (26.67) | 55 (61.11) | 25.56 | 1.08 | (0.69-1.68) | 0.751 |
|  | CCC (182) |  | 10 (5.50) | 73 (40.12) | 99 (54.39) | 25.55 |  |  |  |
| rs8193036 | Asymptomatic (90) | C\|T | 11 (12.22) | 31 (34.44) | 48 (53.33) | 29.44 | 0.74 | (0.49-1.29) | 0.164 |
|  | CCC (182) |  | 18 (9.89) | 55 (30.22) | 109 (59.90) | 25 |  |  |  |
| rs2275913 | Asymptomatic (90) | A\|G | 2 (2.22) | 38 (42.22) | 50 (55.56) | 23.33 | 0.72 | (0.43-1.21) | 0.217 |
|  | CCC (182) |  | 5 (2.75) | 65 (35.71) | 112 (61.54) | 20.6 |  |  |  |

1: minor allele | 2: major allele; alleles are showed in forward strand. MAF: minor allele frequency. OR: odds ratios, L95-U95: confidence intervals of 95% L: lower limit; U: upper limit. Values adjusted by sex and age.

**Table S4-3.** Bolivian cohort

| SNP |  | A1\| A2 | Genotype N (%) | | | MAF | Allele test | | |
| --- | --- | --- | --- | --- | --- | --- | --- | --- | --- |
|  |  |  | 1\|1 | 1\|2 | 2\|2 |  | OR | (L95-U95) | P LogstReg |
| rs4711998 | Asymptomatic (522) | A\|G | 27 (5.17) | 164 (31.41) | 331 (63.4) | 20.88 | 0.96 | (0.65-1.41) | 0.831 |
|  | CCC (100) |  | 3 (3) | 34 (34) | 63 (63) | 20 |  |  |  |
| rs8193036 | Asymptomatic (522) | C\|T | 56 (10.72) | 174 (33.33) | 292 (55.93) | 27.39 | 1.18 | (0.85-1.62) | 0.319 |
|  | CCC (100) |  | 11 (11) | 38 (38) | 51 (51) | 30 |  |  |  |
| rs2275913 | Asymptomatic (522) | A\|G | 16 (3.06) | 117 (22.41) | 389 (74.52) | 14.27 | 1.14 | (0.75-1.71) | 0.543 |
|  | CCC (100) |  | 4 (4) | 24 (24) | 72(72) | 16 |  |  |  |

1: minor allele | 2: major allele; alleles are showed in forward strand. MAF: minor allele frequency. OR: odds ratios, L95-U95: confidence intervals of 95% L: lower limit; U: upper limit. Values adjusted by sex and age.
