# Supplementary material for: Genetic polymorphisms of IL17A associated with Chagas disease: results from a meta-analysis in Latin American populations
Source: Sci Rep. 2020 Mar 19;10:5015. doi: 10.1038/s41598-020-61965-5 (PMC7081280; doi:10.1038/s41598-020-61965-5)
Supplement: Supplementary file 5 — Supplementary information 5. [file 41598_2020_61965_MOESM5_ESM.docx]

*Genetic polymorphisms of IL17A associated with Chagas disease: results from a meta-analysis in Latin American populations*

Mariana Strauss, Miriam Palma-Vega, Desiré Casares-Marfil, Pau Bosch-Nicolau, María Silvina Lo Presti, Israel Molina, Clara Isabel González, Chagas Genetics CYTED Network, Javier Martín, Marialbert Acosta-Herrera

**Table S5.** Genotype and allele distribution for *IL17A* variants in seronegative and Chronic Chagas cardiomyopathy (CCC) individuals

**Table S5-1.** Colombian cohort

| SNP |  | A1\| A2 | Genotype N (%) | | | MAF | Allele test | | |
| --- | --- | --- | --- | --- | --- | --- | --- | --- | --- |
|  |  |  | 1\|1 | 1\|2 | 2\|2 |  | OR | (L95-U95) | P LogstReg |
| rs4711998 | Seronegative (621) | A\|G | 49 (7.89) | 217 (34.94) | 355 (57.16) | 25.36% | 0.93 | (0.75-1.16) | 0.541 |
|  | CCC (565) |  | 31 (5.49) | 200 (35.40) | 334 (59.12) | 23.19% |  |  |  |
| rs8193036 | Seronegative (621) | C\|T | 54 (8.69) | 229 (36.87) | 338 (54.42) | 27.13% | 0.84 | (0.67-1.05) | 0.133 |
|  | CCC (565) |  | 28 (4.96) | 204 (36.11) | 333 (58.94) | 23.01% |  |  |  |
| rs2275913 | Seronegative (621) | A\|G | 27 (4.78) | 175 (30.97) | 363 (64.25) | 19.00% | 1.14 | (0.90-1.44) | 0.298 |
|  | CCC (565) |  | 25 (4.03) | 186 (29.95) | 410 (66.02) | 20.27% |  |  |  |

1: minor allele | 2: major allele; alleles are showed in forward strand. MAF: minor allele frequency. OR: odds ratios, L95-U95: confidence intervals of 95% L: lower limit; U: upper limit. Values adjusted by sex and age.

**Table S5-2.** Argentinian cohort

| SNP |  | A1\| A2 | Genotype. N (%) | | | MAF | Allele test | | |
| --- | --- | --- | --- | --- | --- | --- | --- | --- | --- |
|  |  |  | 1\|1 | 1\|2 | 2\|2 |  | OR | (L95-U95) | P LogstReg |
| rs4711998 | Seronegative (76) | A\|G | 4 (5.26) | 22 (28.95) | 50 (65.79) | 19.74 | 1.47 | (0.92-2.37) | 0.109 |
|  | CCC (182) |  | 10 (5.50) | 73 (40.12) | 99 (54.39) | 25.55 |  |  |  |
| rs8193036 | Seronegative (76) | C\|T | 5 (6.58) | 21 (27.63) | 50 (65.79) | 20.39 | 1.21 | (0.78-1.88) | 0.3894 |
|  | CCC (182) |  | 18 (9.89) | 55 (30.22) | 109 (59.90) | 25 |  |  |  |
| rs2275913 | Seronegative (76) | A\|G | 4 (5.26) | 24 (31.58) | 48 (63.16) | 21.05 | 0.99 | (0.60-1.61) | 0.955 |
|  | CCC (182) |  | 5 (2.75) | 65 (35.71) | 112 (61.54) | 20.6 |  |  |  |

1: minor allele | 2: major allele; alleles are showed in forward strand. MAF: minor allele frequency. OR: odds ratios, L95-U95: confidence intervals of 95% L: lower limit; U: upper limit. Values adjusted by sex and age.
